# Parents' insights after pediatric hospitalization due to rotavirus gastroenteritis in Italy

**DOI:** 10.1080/21645515.2017.1336271

**Published:** 2017-06-13

**Authors:** Federico Marchetti, Volker Vetter, Giorgio Conforti, Susanna Esposito, Paolo Bonanni

**Affiliations:** a GSK, Verona, Italy; b GSK, Wavre, Belgium; c Family Pediatrician, Genova, Italy; d Pediatric Clinic, Università degli Studi di Perugia, Perugia, Italy; e University of Florence, Florence, Italy

**Keywords:** gastroenteritis, hospitalization, Italy, rotavirus, stress

## Abstract

Most of the severe cases of acute gastroenteritis in infants and children under 5 globally are caused by rotavirus infection. There are nearly 15,000 rotavirus gastroenteritis (RVGE) hospitalizations in Italy each year, which could be reduced by available rotavirus vaccines. In addition to the economic and societal burden, RVGE hospitalization could impact families negatively. The aim of this survey was to obtain parents' insights after hospitalization of their child for RVGE. Parents, of 500 children aged 0–5 years, were interviewed about their experience of RVGE hospitalization and asked to rate their stress on different items and overall. Most children (32.6%) were hospitalized aged 12–23 months, and 6.8% were <6 months old. Family pediatricians referred 56.2% of cases to hospital, and 25.8% went based on their parents' decision. During hospitalization, mean parental stress scores (out of 10, with 10 as highest stress) ranged from 6.6 to 8.4. The highest scores were for child malaise (8.42, SD 1.00), vomiting/diarrhea (8.07, SD 0.97), stress for the family in general (7.82, SD 0.90), parental stress (7.68, SD 0.93) and child dehydration (7.18, SD 1.02). The overall stress for the family was graded as ‘high' by 67.2% of parents.

Geographical areas and stress level were related (p = 0.0071), being the “high” stress score not an evenly distributed variable (p < 0.0001). Most children (91.8%) were not vaccinated against rotavirus, as most parents (74.5%) were not aware of vaccination availability. Parental distress due to RVGE hospitalization appears to be significant (93.6% reporting high/medium stress) and there is an important lack of awareness among parents about rotavirus vaccination. More education on RVGE for families in Italy should be warranted.

## Introduction

Rotavirus infection is the primary cause of acute gastroenteritis (AGE) in infants and children under 5 y of age worldwide, and is responsible for most of the severe cases.^1,2^ The disease can be asymptomatic in older children and adults, but incidence is highest in children 6–24 months old, and those <12 months old are at greatest risk of rotavirus gastroenteritis (RVGE) with severe diarrhea, vomiting and fever that can result in dehydration and the need for hospitalization.^3^ Furthermore, RVGE exerts an important negative impact on the healthcare system, due to medical resource use and costs.^3^

In Italy, there are approximately 15,000 hospitalizations each year due to RVGE.^4^ An analysis of hospital discharge data from 2005 to 2012 estimated the hospitalization rate for RVGE in children under 6 y old to be 146 and 150 per 100,000 children, as primary and secondary diagnosis, respectively.^2^ In the Lombardy region, 50.8% of AGE hospitalizations were found to be due to rotavirus and a further 14% had a possible link to the virus.^5^ Both studies found a peak in hospitalization among children under 2 y old. An observational study performed in the community in Italy documented that a third of infants and a fifth of young children under 5 y old were assisted by a single family pediatrician for AGE, a more frequent condition than influenza-like illness in this population. Rotavirus was often responsible for the condition, accounting for up to 53.9% and 49.1% of cases in spring 2005 and winter 2004–5, respectively.^6^ In summary, rotavirus was found to be responsible for nearly 50% of AGE in both hospital and community settings.^5,6^ In Italy, RVGE exerts a consistent economic impact and is responsible for over €30 million in direct medical costs and around €112 million in indirect costs each year.^7^

Rotavirus vaccines, available since 2006, have been recommended for all children by the World Health Organization (WHO) since 2009 and have been successfully implemented, reducing RVGE incidence and costs, in several European countries and the USA in the last 10 y.^2,8^ Recently, a natural experiment took place in Northern Ireland, which has implemented routine rotavirus vaccination, and the Republic of Ireland, with no rotavirus vaccination program. In Northern Ireland, 2 y after introducing vaccination, the incidence of rotavirus infection was reduced by 54% compared with the pre-vaccination era, AGE notifications in the highest risk group (< 2 y old) decreased by 53% and AGE hospitalizations decreased by 40% in children <5 y old. By contrast, in the Republic of Ireland, the incidence of AGE was found to increase in the same time period.^9^ On top of their effectiveness, RVGE vaccination programs proved to be long-lasting. In Belgium, 7 y after introducing routine rotavirus vaccination, rotavirus infection had decreased by 79% in <2 year-olds and RVGE hospitalization by 87% compared with the pre-vaccination period. In addition, there was a 50% decrease in cases ≥ 10 y old, most likely due to herd immunity.^10^

Rotavirus vaccines are available for a co-payment in most Italian regions,^11^ however, a key barrier to vaccination remains the lack of awareness of the disease burden. Sicily was the first Italian region to have implemented universal rotavirus vaccination, in January 2013, and witnessed a decrease of 35% and 47% in hospitalization rates for children aged <59 months and <12 months, respectively. These reductions occurred in the first year of vaccination despite only 35% of children <1 y old being fully vaccinated at the time.^12^

RVGE hospitalization may also exert a direct negative effect on families. Therefore the aim of this survey was to explore parents' insights after hospitalization of their child for RVGE.

## Results

In December 2015, a sample of nearly 800 parents were identified from the database as having a child aged 0–5 y old hospitalized in the last 5 y for RVGE. They were contacted to participate in the survey with the aim of obtaining 500 participants equally distributed over the country. There were 500 parents that accepted to take part in the survey and were interviewed regarding their experience of having a child aged 0–5 y old hospitalized due to RVGE. The participants were distributed by region as follows; 15% northwest, 21% northeast, 22% central and 42% south and islands. At the time of the interview, two thirds of parents were aged 30–39 years, and, 45.2% were female. (See Table 1 for demographic characteristics.)

**Table 1.** Participants' demographic characteristics and location.

Characteristic	Number n	Percent %
**Regional distribution**		
Northwest	75	15%
Northeast	105	21%
Central	110	22%
South & Islands	210	42%
**Parents' age at interview**		
≤ 29 y	111	22.2%
30–34 y	162	32.4%
35–39 y	166	33.2%
≥ 40 y	61	12.2%
**Parents' gender**		
Female	226	45.2%
Male	274	54.8%
**Parents' education level**		
Primary school	125	25.0%
Middle school	167	33.4%
High school	171	34.2%
Degree	37	7.4%
**Parents' profession**		
Unemployed	81	16.2%
Laborer	72	14.4%
Employee	129	25.8%
Tradesman / Craftsman	117	23.4%
Housewife	51	10.2%
Freelancer	40	8.0%
Entrepreneur / Business owner	10	2.0%

**Figure 1.**
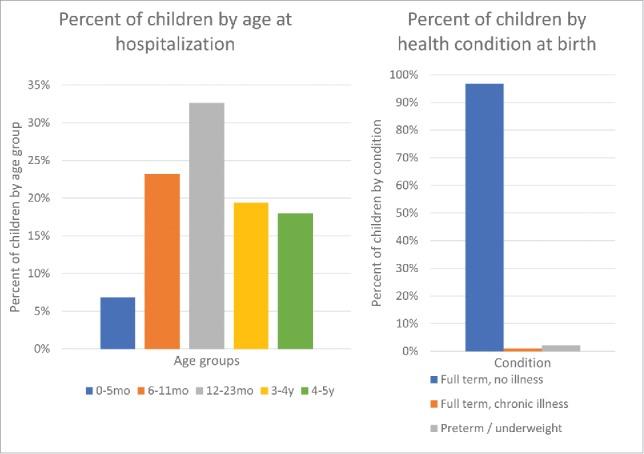
Child's age at hospitalization and health condition at birth.

At the time of hospitalization, the majority of children (32.6%) were aged 12–23 months, and 6.8% were <6 months of age. The great majority of hospitalized children (96.8%) were born at full term without any chronic illness, 2.2% were born preterm (< 38 weeks) or underweight (< 2500 g), and 1% were born at full term with a chronic illness. (Fig. 1)

Of the 500 children hospitalized, 56.2% were referred by their family pediatrician, 25.8% went to hospital based on their parents' own decision, and a further 8.8% and 7.0% went based on advice from other mothers and relatives/friends, respectively. The most common reasons for hospitalization were acute diarrhea (cited by 47.2% of parents), and, poor general condition and dehydration (29.1%). Other reasons included persistent vomiting (13.4%), fever (9.4%) and convulsions (1.0%).

**Figure 2.**
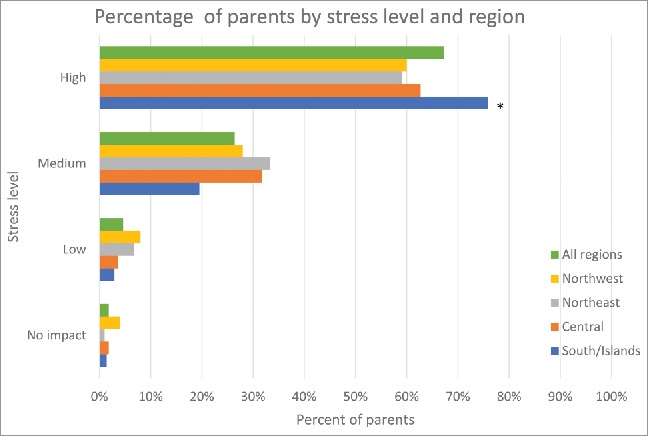
Percentage of parents by overall stress level and region.

During hospitalization of their children, parents' stress scores (mean scores out of 10, standard deviation [SD]) ranged from 6.6 to 8.4 for the different items scored. The highest scores were related to child malaise (8.42, SD 1.00), vomiting/diarrhea (8.07, SD 0.97), stress for the family in general (7.82, SD 0.90), parental stress (7.68, SD 0.93) and child dehydration (7.18, SD 1.02). Items with lower stress scores included, days of job loss/other loss of earnings, child weight loss, limited time for managing the home/family, and, the need to ask for help/support from other people. The overall stress for the family was rated as ‘high' by 67.2% of parents, with some variations across the country (Fig. 2). Overall, geographical areas and stress were related (p = 0,0071), being the “high” stress score not an evenly distributed variable (p < 0.0001), while for ‘medium' (p = 0.0879), ‘low' (p = 0.8432) and ‘no stress' (p = 0.8846) categories an even distribution was found out.

Almost all of the parents (91.8%) reported that their children had not received the rotavirus vaccination before hospitalization; 74.5% were not aware of the availability of the vaccine while 19% were aware. These parents had decided against vaccination primarily because it was not a mandatory vaccine, it was only available for a fee, or, it was not available at the Local Health Unit.

Following hospitalization of their child, however, the majority of participants would ‘strongly recommend' (79.8%) and ‘possibly recommend' (10.8%) rotavirus vaccination to other mothers/parents. According to the respondents' judgment, the most relevant sources of information (mean scores out of 10, SD) when deciding to vaccinate their child were the family pediatrician (7.95, SD 1.05), the internet (7.76, SD 1.14), other mothers/parents (7.14, SD 1.13); other factors had less influence such as; relatives/friends (5.98, SD 0.93), Local Health Unit (5.64, SD 0.95), TV/radio (5.25, SD 0.92), baby courses (5.23, SD 1.00), and, the school (5.22, SD 0.96).

## Discussion

This national survey of parents provided insights on their experience of having a child <5 y old hospitalized for RVGE. Among 500 hospitalized children, just over half were referred by their pediatrician, and, most were admitted for acute diarrhea or poor general condition and dehydration. Mean parental stress scores out of 10 ranged from 6.6 (SD 0.87) to 8.4 (SD 1.00), and were highest for child malaise and vomiting/diarrhea. Two thirds of parents rated the overall experience as ‘high' stress. Although RVGE is a vaccine-preventable condition, more than 90% of children were not vaccinated, most of them (74.5%) due to lack of awareness of the rotavirus vaccine.

More than 90% of hospitalized children from the survey were born at full term, with no chronic illness; this figure confirms the importance of including all newborns (universal mass vaccination) in the rotavirus vaccination programs, as recommended in many countries worldwide, and not to restrict it to the so-called “at risk” children.

The literature on family stress linked to hospitalization of children focuses mainly on studies in chronic or life-threatening conditions. Data were limited on acute conditions such as RVGE and none of these studies were specifically designed to collect insights from families. In the REVEAL study, a large prospective observational study conducted in 7 European countries including Italy, parents rated their stress relating to the RVGE on a visual analog scale, in hospitals or outpatient settings. This study found relatively high parental stress scores (ranging from 4.5 to 8.9 out of 10).^13^ Despite being based on a somewhat smaller sample, these findings are comparable to the findings of our hospital survey albeit slightly lower, which may be explained by lower stress levels in the outpatient versus hospital setting. In another prospective observational European study of RVGE in primary care, parents completed a specifically-designed and validated RVGE questionnaire assessing, among other things, parental distress, worry due to child's symptoms, and, daily activities. The study found an adverse effect on activities of daily living in addition to increased worry and distress that increased with worsening severity of symptoms in the child. Although this was an uncontrolled study, the authors cite other studies in Europe and North America, in both hospital and outpatient settings, showing a similar negative impact of RVGE on parents' daily activities, work, worry and distress scores, and generally high levels of stress associated with the condition.^14^ A recent literature review of the psychosocial impact of RVGE found studies had used a range of generic quality of life instruments including the EuroQol (EQ-5D) and Health Utilities Index 2 (HUI2). The main impact of the disease on parents' quality of life was due to ‘parental worry due to symptoms'. Other aspects of parents' quality of life that were impacted by RVGE included daily activities, social interactions, pain/discomfort, and, concern about managing care of the child and other household activities. One study in Spain found greater worry levels in parents of children with RVGE vs. AGE.^15^ Data from the REVEAL study, found mothers experienced high stress due to RVGE, with greater stress levels for hospitalized children vs. those seen in primary care or admitted to the emergency department.^13^

Family pediatricians play a major role in either sending families to the hospital for RVGE, or in counseling on rotavirus vaccination, and this was confirmed in the survey results. Good communication to families is essential to increase their awareness of RVGE and the value of vaccination. Vaccine hesitancy, defined as a ‘delay in acceptance or refusal of vaccination', results in poor control of the disease and in its subsequent burden to society. Recent research on the topic in Italy has concluded that communication by healthcare providers must involve dialog with the public, taking into account the influence of (social) media and other sources of information that could affect parents' decisions about vaccination. Successful delivery of evidence-based information can require training and shifting of attitudes among healthcare professionals, and must be done with the collaboration of family doctors, health services and the media.^16^

The aim of this survey was to gain insights from parents' experience of RVGE hospitalization, through speaking to parents from across Italy. The main limitations of this market (social) research study include the use of a convenience sample rather than randomly selected subjects, the lack of a control group, the use of a simple scoring system rather than a validated quality of life questionnaire, and the need for some parents to answer questions based on recalling their experience up to 5 y ago. Nevertheless, to the best of our knowledge, this represents the first attempt to capture parents' insights in Italy on distress following RVGE hospitalization, which has turned out to be significant (93.6% reporting high/medium stress).

In conclusion, many of the parents interviewed were not aware of the rotavirus vaccination, and results showed high parental stress levels associated with RVGE hospitalization of their children. This outcome suggests there is a need for an RVGE educational program for families in Italy.

## Patients and methods

### Subjects

The survey was performed as a market (social) research, on behalf of GlaxoSmithKline S.p.A, Italy, by *Datanalysis*, a health research institute, that developed a searchable electronic database of patients or families over the last 30 y. The database was searched using the keyword “rotavirus” and going back for a period of 5 y. The aim was to recruit at least 500 subjects from across Italy, given the time and resources available for the survey. Parents with a child aged 0–5 y old hospitalized in the last 5 y for RVGE were identified from the database and contacted by telephone for participation in the survey. Due to the qualitative nature of the survey, no control group was needed.

### Study design

Telephone interviews were conducted with parents by interviewers trained to conduct phone interviews with a questionnaire. The interviews consisted of 13 questions formulated for this survey, some of which required parents to select an answer from a list of possible options. The questionnaire comprised the following topics; questions 1–4 ascertained the child's current age and health status at birth, confirmed the hospitalization diagnosis (i.e., RVGE) and age at hospitalization; questions 5–6 ascertained the reason for (e.g., symptoms) and method of (e.g., referral) hospitalization; questions 7–8 required parents to rate (score from 1 to 10) their stress for specific items (e.g., impact on the child, the family, or work) and their overall experience; and questions 9–13 related to decisions around vaccination (i.e., prior vaccination and reasons for vaccinating or not vaccinating, given their experience would they recommend vaccination to others, and, what influences vaccination decisions). (See Supplemental material 1.)

The items in question 7 that parents had to score their stress levels on were determined following a review of the literature.

### Analysis

Data collected from the questionnaire were analyzed by region (Northwest, Northeast, Central and South & Islands) and overall for Italy. Descriptive statistics (i.e., numbers and percent of responders overall and by subgroup, computed mean scores and SD), were analyzed for each question using IBM SPSS Statistics software v11.0.

For stress comparison, regions were considered as nominal categorical variable and stress level to be an ordinal categorical variable, as the values of stress level are in increasing order in categories. The overall effect that regional distribution exerted on stress levels was evaluated by a Cochran-Mantel-Haenszel Chi-Squared test. To evaluate if there was an even distribution within each stress level among the regions, a one-way Chi-Squared tests was selected. If the expected number of respondents were too small (less than 5), an exact Chi-Squared test had to be used.

### Complying with ethics of experimentation

This market research survey did not require a formal Ethics review committee. This study was performed in compliance with the Law Decree n. 196/2003, article 24 (Code for the protection of personal data).

### Consent

This survey did not require a specific personal consent, as subjects were retrieved from a pre-existing database where initial general consent was obtained at the time of subscription.

## Supplementary Material

2017HV0090R-s02.docx
