# Supplementary material for: Parents' insights after pediatric hospitalization due to rotavirus gastroenteritis in Italy
Source: Hum Vaccin Immunother. 2017 Jun 13;13(9):2155–9. doi: 10.1080/21645515.2017.1336271 (PMC5612036; doi:10.1080/21645515.2017.1336271)
Supplement: 2017HV0090R-s02.docx [file khvi-13-09-1336271-s001.docx]

**Supplemental material 1: Questionnaire used for the survey (translated from Italian)**

Question 1: Do you have any children aged a few months to ≤5 years old?

Question 2: If yes, have you ever had a child in this age group hospitalized for RVGE?

Question 3: If yes, how old was your child when they were hospitalized?

Question 4: Was your child born;

1. At full term with no chronic illness
2. At full term with a chronic illness
3. Pre-term / underweight

Question 5: For which of the following reasons did you decide to hospitalize your child;

1. Acute diarrhea
2. Persistent vomiting
3. Poor general condition and dehydration
4. Fever
5. Convulsions

Question 6: Who suggested you bring your child to the hospital;

1. Decided yourself
2. Family pediatrician
3. Another medical professional
4. Relatives/friends
5. Other mothers

Question 7: Regarding the time spent in hospital, can you rate your level of concern about the following aspects (scale of 1 to 10, with 10 as highest stress)

1. Child’s dehydration
2. Child’s weight loss
3. Child’s vomiting and/or diarrhea
4. Stress/worry about the child
5. Stress/worry about the parents
6. Stress/worry about the family in general
7. Limited time available for managing home/family
8. Loss of work days
9. Need to ask others for help or support

Question 8: In summary, how would you rate the overall experience of hospitalization for you and your family, in terms of level of discomfort/stress?

1. No impact
2. Low stress
3. Medium stress
4. High stress

Question 9: Was your child vaccinated against rotavirus prior to hospitalization?

Question 10: If you answered ‘No’ to question 9; were you aware of the availability of rotavirus vaccines?

Question 11: If you answered ‘Yes’ to question 10; why did you decide not to vaccinate your child?

1. Not an obligatory vaccine
2. Fear of vaccination
3. Vaccine was only available for a fee
4. Vaccine was not available at the Local Health Unit
5. Pediatrician did not inform you or recommend it

Question 12: Given this experience, would you recommend rotavirus vaccination to other mothers/parents?

1. Absolutely yes
2. Possibly yes
3. Possibly not
4. Absolutely not

Question 13: Thinking about vaccination in general today, rate (1-10) the most influential sources of information to help you decide whether to vaccinate your child?

1. Family pediatrician
2. The internet
3. Other mothers/parents
4. Relatives/friends
5. Local Health Unit
6. TV/radio
7. School
8. Baby courses
